# Creatine Supplementation Dose and Duration Are Not Associated with Increased Side Effects: A Structured Review and Study-Level Dose–Response Analysis of Randomized Controlled Trials

**DOI:** 10.3390/sports14040137

**Published:** 2026-04-01

**Authors:** Drew E. Gonzalez, Broderick L. Dickerson, Kelly Hines, Jisun Chun, Adriana Gil, Scott C. Forbes, Darren G. Candow, Richard B. Kreider

**Affiliations:** 1 Exercise and Sport Nutrition Laboratory, Department of Kinesiology and Sport Management, Texas A&M University, College Station, TX 77843, USA; dickersobl5@tamu.edu (B.L.D.); chunjs3112@tamu.edu (J.C.); 2 Occupational, Performance, and Nutrition Laboratory, Department of Kinesiology, Sam Houston State University, Huntsville, TX 77341, USA; rbkreider@tamu.edu; 3 Department of Kinesiology, School of Natural Sciences, St. Edwards University, Austin, TX 78704, USA; 4 Department of Exercise and Nutrition Sciences, University at Buffalo, Buffalo, NY 14260, USA; kellyhin@buffalo.edu; 5 Department of Obstetrics and Gynecology, Baylor College of Medicine, Houston, TX 77030, USA; adriana.gil@bcm.edu; 6 Department of Physical Education Studies, Faculty of Education, Brandon University, Brandon, MB R7A 6A9, Canada; forbess@brandonu.ca; 7 Faculty of Kinesiology and Health Studies, University of Regina, Regina, SK S4S 0A2, Canada; darren.candow@uregina.ca

**Keywords:** creatine, supplement safety, tolerability, side-effect profile, ergogenic aids, nutritional supplements

## Abstract

There are concerns that high-dose and/or long-term creatine monohydrate supplementation (CrM) leads to greater side effects (SEs) compared to placebo. This analysis investigated whether the dose or duration of CrM was associated with SEs. Data from trials involving more than 12,800 participants within CrM and placebo study arms of 684 randomized clinical trials were analyzed. SEs were combined into categories and total absolute dose and CrM duration were grouped into tertiles (low, moderate, and high). Crosstabs with chi-square tests were used to compare the prevalence of SEs across CrM dose and duration tertiles. Logistic regression models, adjusted for biological sex, age, and population categories, were used to test dose and duration as continuous predictors. Across 684 randomized controlled trials, reported SEs were infrequent. Although dose and duration tertiles were statistically associated with study-level side effect reporting, the effect sizes were uniformly small, events were infrequent, and the reported symptoms were primarily mild and nonspecific. No consistent exposure–response pattern indicative of clinically meaningful risk was observed. Adjusted logistic regression and frequency-based analyses showed no consistent dose- or duration-dependent increase in SE risk, with placebo groups often reporting similar or greater SE frequencies at the study-reporting level. CrM appears to be well-tolerated and, at the study-level, does not increase the risk of gastrointestinal, renal, liver, musculoskeletal, or other SEs compared to placebo, even at high doses or longer durations.

## 1. Introduction

Creatine is one of the most well-studied nutritional ergogenic aids for recreational and trained athletes alike [1]. Creatine supplementation boosts cellular energy by increasing intramuscular phosphocreatine stores [1]. This enhancement allows for faster ATP resynthesis during high-intensity, short-duration activities. As a result, creatine supports better performance in repeated high-intensity efforts and resistance training. Multiple randomized controlled trials have confirmed that creatine leads to greater strength, higher power output, improved repeat sprint or high-intensity performance, and the ability to handle higher training volumes over time. These performance-enhancing effects explain its popularity among athletes and in clinical settings [1]. Recently, research on the applications of CrM in females [2], youth and adolescent athletes [3], and clinical populations [4] has increased. Interestingly, although numerous studies demonstrate that CrM is safe [1], myths and misconceptions about its safety profile persist [5]. Importantly, this view draws on earlier scholarly discussions that highlighted ongoing safety concerns in both public and clinical debates, rather than aiming to dismiss legitimate scientific evidence inquiry. Collectively, the available evidence does not support many of the negative claims commonly attributed to the use of CrM; however, these myths and misconceptions continue to persist among the general public and have even influenced policy and legislative discussions [5]. Although policy considerations are often multifactorial, clarifying the randomized trial evidence base remains important for informing evidence-based decision-making and for evaluating whether consistent exposure–response patterns are evident within the existing literature.

Since the 1970s, over 650 peer-reviewed clinical trials, mostly involving CrM, have examined creatine supplementation [5]. Previous work has demonstrated that these studies, involving more than 12,800 participants, used doses of up to 30 g daily for up to 14 years across diverse populations, from infants to the very elderly, in both healthy and clinical settings [5]. Furthermore, no clinical side effects were reported, and minor side effects were rare and not statistically significantly more frequent than in over 13,500 placebo cases [5]. This includes studies of children and adolescents (<18 years), young adults (19–45 years), middle-aged adults (46–65 years), and older adults (>65 years). Additionally, analysis of more than 28.4 million side effect reports from the United States of America (U.S.), Canada, Australia, and Europe over 50 years, using SIDER 4.1, shows that creatine is mentioned in only about 0.0007% of reports, despite billions of doses administered globally in the last 30 years [5]. Although side-effect reports do not establish causality, the near absence of reports worldwide supports the clinical findings that creatine is safe for individuals of all ages. While spontaneous reporting systems cannot definitively establish causality and may be affected by reporting biases, their observations generally align with findings from controlled clinical study trials. For clarity, in this manuscript, the term “side effects” refers to any adverse event reported within a randomized controlled trial arm, regardless of causality assessment. This differs from clinically confirmed adverse outcomes as the reporting prevalence indicates whether at least one participant reported a symptom during the study, not necessarily establishing causality or medical confirmation event.

Collectively, the existing body of evidence demonstrates that CrM has been extensively evaluated for safety across a wide range of doses, durations, and populations, with clinical trials consistently reporting no serious side effects and only infrequent, mild side effects, even under higher-dose or longer-term supplementation protocols [1]. On the basis of this evidence, CrM has been designated as Generally Recognized as Safe (GRAS) by the U.S. Food and Drug Administration [6] and remains the only form of creatine broadly approved for commercial sale across major international regulatory jurisdictions, including North America, Europe, Asia, and Australia. Despite this robust safety record, anecdotal reports and unsupported claims regarding side effects, renal risk, and the purported superiority of alternative creatine formulations continue to circulate widely, particularly in online and popular media sources. The ongoing debate over safety perceptions underscores the importance of continued, transparent evaluation of the evidence base. Given the breadth of available data and the continued interest in exposure–response relationships, a structured and transparent synthesis of dose- and duration-dependent side effect reporting is warranted. Accordingly, the purpose of the present analysis was to directly address these concerns by systematically evaluating whether total CrM dose or duration predicts the likelihood of side effects, stratified by physiological system, thereby providing a more granular and clinically relevant assessment of CrM safety.

## 2. Materials and Methods

### 2.1. Comprehensive Literature Review Method and Study Sample

**Figure 1:**
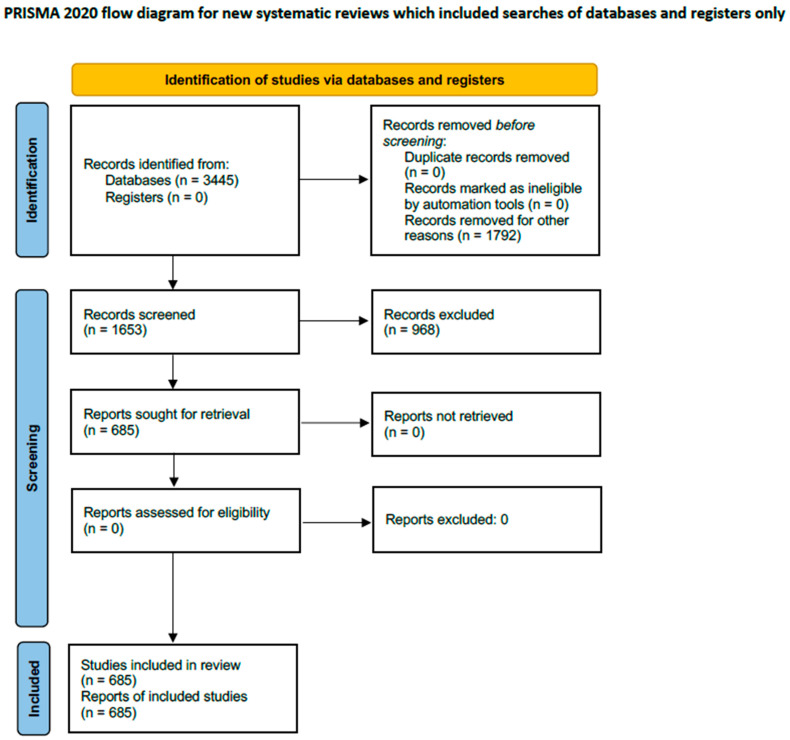
PRISMA-informed flow diagram of study identification and screening. A comprehensive PubMed search using the keywords “creatine” and “supplementation” identified 3445 records. After removing publications that did not involve human clinical trials (animal studies, literature reviews, meta-analyses, patents, and unrelated publications), 1653 records remained for screening. From these, 685 randomized controlled trials investigating creatine supplementation in humans were included in the adverse event analysis. Records removed prior to screening included animal studies, literature reviews, meta-analyses, patents, and unrelated publications.

Previously, we conducted a comprehensive PubMed literature review using the keywords “creatine” and “supplementation” [5]. We compared the search algorithm results with a comprehensive database created by the Principal Investigator, comprising 3445 unique references. We excluded studies that did not focus on creatine supplementation (*n* = 539), involved animal models (*n* = 1186), or were literature reviews (*n* = 496), meta-analyses (*n* = 98), patents (*n* = 6), or other unrelated publications (*n* = 6). After this screening, 1113 articles remained, including 685 randomized controlled clinical trials in humans with data from 1337 participants comparing creatine with placebo. These were subsequently reviewed and analyzed for side effects. This analysis is a comprehensive collection of all peer-reviewed randomized controlled trials on creatine monohydrate supplementation. Since the goal was to include the entire body of RCT evidence without selectively choosing studies based on outcomes or methods, traditional systematic review steps like duplicate screening, formal risk-of-bias assessment, and protocol registration were not necessary. Instead, all eligible RCTs were documented and organized for adverse event data extraction. Although the database aimed to capture the complete body of RCT evidence rather than perform a traditional outcome-based systematic review, elements of systematic review methodology were applied to enhance transparency. The search strategy, screening process, and eligibility criteria are summarized following PRISMA guidelines in Figure 1.

### 2.2. Study Summaries and Categorization

After identifying human clinical trials on creatine supplementation, each eligible study was independently reviewed. Key methodological details and outcomes were extracted and compiled into a comprehensive, searchable spreadsheet. This database includes bibliographic information, publication year, sample size per study arm, study design, intervention duration, absolute and body-mass-adjusted creatine doses, participant demographics, primary outcomes, and any reported side effects for both the creatine and placebo conditions. Side effects reported across studies were compiled into a standardized list of 35 side-effect indicators. For each trial, the presence or absence of each indicator was recorded as a binary yes/no outcome, facilitating a comparison of side effects between the creatine and placebo groups. When side effects were mentioned, their frequencies were documented separately for each supplement condition. If a specific side effect was not reported in a study, it was assigned a value of 0. These data were then combined to calculate the total number of side effects reported per study. Each trial was categorized by supplementation group (creatine or placebo), participant sex (mixed, male-only, female-only, or unspecified), age group (children/adolescents under 18, young adults 18–45, middle-aged adults 45–65, or older adults over 65), training status (clinical populations with physical or cognitive conditions, untrained, recreationally active, trained, athletic, or military groups), health status (healthy or clinical), and clinical classification (e.g., cardiometabolic, cardiopulmonary, neurological, musculoskeletal, renal, or other conditions). These classifications were used to describe the participant demographics, dosing methods, reporting of side effects (whether present or absent), and the occurrence of specific side effects in the creatine and placebo groups. When demographic details, such as body mass, were unavailable, population-based average values were used to estimate relative creatine intake. Although the RCT database was initially assembled for descriptive safety summaries, this analysis now includes new exposure-normalized variables and employs regression-based modeling, which were not part of the previous publication [5]. Because adverse events were not consistently reported across trials, the absence of reporting was coded as ‘not reported’ rather than assuming the absence of occurrence.

### 2.3. Statistical Analysis

All statistical analyses were conducted using IBM SPSS Statistics for Windows (Version 29.0.2.0; IBM Corp., Armonk, NY, USA). Analyses were performed at the study level, with each randomized controlled trial (RCT) considered a single observational unit. Statistical significance was defined as *p* ≤ 0.05. Since the data are at the study level, the logistic regression results indicate differences between studies in reporting, rather than in individual participant risk. Consequently, findings are interpreted as variations in the likelihood that an RCT reports side effects, rather than as participant-level differences in incidence.

#### 2.3.1. Exposure Categorization

Total absolute creatine exposure (g) and total supplementation duration (days) were computed for each RCT. Both variables were then divided into tertiles (low, moderate, and high) according to the 33rd and 66th percentiles of their respective distributions. This categorization was selected to accommodate the substantial heterogeneity in dosing strategies and supplementation durations across trials while allowing for interpretable comparisons across exposure levels. Given the wide variation in dosing protocols across studies, the tertile approach provides a pragmatic framework for summarizing patterns of adverse event reporting rather than modeling precise dose–response relationships.

#### 2.3.2. Side Effect Analyses

The presence or absence of any reported side effect, as well as side effects within each physiological category, was recorded as a binary outcome (yes/no) at the study level. Cross-tabulations with Pearson chi-square tests were used to examine differences in side effect prevalence across dose and duration tertiles. Effect sizes were measured using Cramér’s φ (φc), with values of 0.10, 0.30, and 0.50 indicating small, medium, and large effects, respectively. For clarity, prevalence estimates are presented as percentages within dose- or duration-based tertiles, indicating the likelihood that a study in a given exposure category reported at least one side effect.

#### 2.3.3. Logistic Regression Analyses

Binary logistic regression was employed to estimate the odds of a study reporting at least one side effect. All models were adjusted for predefined covariates, including supplementation group (creatine vs. placebo), biological sex, age group, population/fitness level, and health status (healthy vs. clinical). Three model sets were used: (1) covariates-only models, which examined the association between supplementation group and individual side effects while adjusting for study characteristics; (2) dose- and duration-added models, which incorporated tertiles of total dose and duration alongside covariates to assess whether exposure levels influenced side effect reporting; and (3) tertile-only sensitivity models, which included dose or duration tertiles without continuous exposure variables to test the robustness of categorical exposure effects. Odds ratios (ORs) with 95% confidence intervals (CIs) are provided. Some odds ratios could not be reliably estimated due to the low frequency of many side effects resulting from sparse data or quasi-complete separation. For outcomes with few events or wide confidence intervals, the results are presented descriptively rather than as definitive conclusions. Sparse event distributions can lead to unstable logistic regression estimates, especially when adjusting for covariates, so model outputs were carefully assessed by examining the confidence intervals and potential issue separation.

#### 2.3.4. Frequency-Based Analyses

Beyond prevalence-based analyses, the overall frequency of reported side effects per study was considered as a continuous measure. Generalized linear models (GLMs) were used to compare the total count of side effects across dose and duration tertiles, supplementation groups, and their interactions. Initially, multivariate models were assessed using Wilks’ Lambda, followed by univariate analyses and pairwise comparisons at the 95% confidence level. Effect sizes are reported as partial eta squared (η^2^). When significant omnibus effects were identified in the GLM analyses, pairwise comparisons were carried out using Fisher’s Least Significant Difference (LSD) procedure at a 95% confidence level. The LSD method was chosen because tertile comparisons were predefined and focused on structured dose- and duration-based contrasts, rather than exploratory tests across many independent endpoints. Due to the extensive and varied evidence base, a more conservative family-wise correction (such as Bonferroni) was not used, as such adjustments could greatly increase Type II errors and hide potentially important exposure-related patterns. Notably, the interpretation of pairwise outcomes focused on effect size, confidence interval width, and consistency across supporting analyses, rather than on isolated results’ *p*-values.

## 3. Results

### 3.1. Overall Study Prevalence of Reported Side Effects

Among all RCTs (*n* = 684), the overall prevalence of reported side effects, assessed across physiological systems/categories and recorded as yes/no, was low among participants enrolled in the creatine study arms (Table 1). Out of all included RCTs, 93 (13.6%) recorded at least one participant having reported a side effect following supplementation with creatine, while 591 (86.4%) did not have any reported side effects within the creatine study arm. In the low-dose tertile, 22 of 251 RCTs (8.8%) reported having at least one recorded side effect compared to 24 of 207 (11.6%) in the moderate-dose tertile and 47 of 226 (20.8%) in the high-dose tertile. Chi-square analysis revealed a statistically significant association between dose tertile and side-effect occurrence (χ^2^(2) = 15.667, *p* < 0.001, φc = 0.151), wherein there was an increase in recorded side effects (or at least one noted) within RCTs across the low- to moderate- to high-dose tertiles. When analyzed by tertiles of total days of supplementation, the occurrence of side effects increased similarly across the RCTs. In the low-duration tertile, 19 of 270 RCTs (7.0%) reported at least one side effect compared with 31 of 199 (15.6%) in the moderate-duration tertile and 43 of 215 (20.0%) in the high-duration tertile. Chi-square analysis revealed a statistically significant association between duration tertile and side-effect occurrence (χ^2^(2) = 18.058, *p* < 0.001, φc = 0.162).

### 3.2. Study Prevalence of Side Effects Reported by Physiological Classification

Table 2 summarizes the study-level prevalence of reported side effects by physiological classification across tertiles of total creatine dose and supplementation duration, showing that side effects were infrequently reported overall and, when present, were more commonly observed in higher-exposure tertiles, which showed small effect sizes.

#### 3.2.1. Gastrointestinal Issues

Among all included RCTs, only 68 (9.9%) reported gastrointestinal-related side effects among participants enrolled in the creatine study arms, whereas 616 (90.1%) reported none. The occurrences of side effects reported within these RCTs increased slightly across tertiles of total absolute creatine dose, wherein 14 (5.6%) of 251 RCTs in the low-dose tertile reported any side effect compared to 17 of 207 (8.2%) in the moderate-dose tertile and 37 of 226 (16.4%) in the high-dose tertile. Chi-square analysis revealed a statistically significant association between dose tertile and the occurrence of side effects (χ^2^(2) = 16.467, *p* < 0.001, φc = 0.155). When analyzed by tertiles of total days of supplementation, the occurrence of gastrointestinal-related side effects increased across studies. In the low-duration group, 14 of 270 RCTs (5.2%) reported gastrointestinal-related side effects compared to 22 of 199 (11.1%) in the moderate-duration group and 32 of 215 (14.9%) in the high-duration tertile. Chi-square analysis showed a statistically significant association between the duration tertile and the occurrence of SEs (χ^2^(2) = 12.963, *p* = 0.002, φc = 0.138).

#### 3.2.2. Renal- or Urinary-Related Issues

Among all included RCTs, only three (0.4%) reported side effects related to renal- or urinary issues, whereas 681 (99.6%) reported none. These side effects were reported only in the high-dose tertile, with three (1.3%) of the 226 RCTs noting them. Chi-square analysis found a statistically significant association between dose tertile and side-effect occurrence (χ^2^(2) = 6.106, *p* = 0.047, φc = 0.094), whereas only the high-dose group had RCTs reporting these side effects. When analyzed by tertiles of total days of supplementation, no renal or urinary issues were reported in the low- and moderate-duration tertiles, whereas three of 215 RCTs (1.4%) reported these issues in the high-duration group. Chi-square analysis showed a statistically significant association between the duration tertile and the occurrence of SEs (χ^2^(2) = 6.573, *p* = 0.037, φc = 0.098), with three studies reporting SEs in the longer-duration tertile.

#### 3.2.3. Liver-, Metabolic-, or Nutrition-Related Issues

Among all included RCTs, only one (0.1%) reported side effects related to liver, metabolic, or nutritional issues, whereas 683 (99.9%) reported none. These side effects were only reported in the high-dose tertile, with one (0.4%) of the 226 RCTs noting them. Chi-square analysis found no association between dose tertile and side-effect occurrence (χ^2^(2) = 2.030, *p* = 0.362, φc = 0.54). When analyzed by tertiles of total days of supplementation, there were no reported side effects related to liver, metabolic, or nutritional issues in the low- and moderate-duration tertiles, whereas one of 215 RCTs (0.5%) reported these issues in the high-duration tertile. Chi-square analysis showed no significant association between duration tertiles and the occurrence of side effects (χ^2^(2) = 2.185, *p* = 0.335, φc = 0.57).

#### 3.2.4. Musculoskeletal Issues

Among all included RCTs, only 20 (2.9%) reported musculoskeletal-related side effects among participants, whereas 664 (97.1%) reported none. The low-dose tertile had these effects in one of 251 RCTs (0.4%), whereas the moderate-dose tertile had five of 207 (2.4%) reporting side effects in this category compared to 14 of 226 (6.2%) for the high-dose tertile. Chi-square tests showed a statistically significant association between dose tertile and the occurrence of side effects (χ^2^(2) = 14.346, *p* < 0.001, φc = 0.145). When analyzed by tertiles of total days of supplementation, no musculoskeletal side effects were reported in the low-duration tertile, whereas eight of 199 (4.0%) and 12 of 215 (5.6%) reported these issues in the moderate- and high-duration tertiles, respectively. Chi-square analysis showed a statistically significant association between duration tertile and side-effect occurrence (χ^2^(2) = 14.324, *p* < 0.001, φc = 0.145).

#### 3.2.5. Neurological- or Vestibular-Related Issues

Among all included RCTs, only 17 (2.5%) reported neurological- or vestibular-related side effects among participants, whereas 667 (97.5%) reported none. The occurrences of side effects reported within these RCTs varied across tertiles of total absolute creatine dose, wherein three (1.2%) of 251 RCTs in the low-dose tertile reported these side effects, compared to three of 207 (1.4%) in the moderate-dose tertile and 11 of 226 (4.9%) in the high-dose tertile. Chi-square analysis showed a statistically significant association between dose tertile and the occurrence of side effects (χ^2^(2) = 7.931, *p* = 0.019, φc = 0.108). When analyzed by tertiles of total days of supplementation, one (0.4%) of 270 RCTs reported a neurological- or vestibular-related side effect in the low-duration tertile, whereas seven of 199 (3.5%) and nine of 215 (4.2%) reported these effects in the moderate- and high-duration tertiles, respectively. Chi-square analysis showed a statistically significant association between duration tertile and side-effect occurrence (χ^2^(2) = 8.424, *p* = 0.015, φc = 0.111).

#### 3.2.6. Sleep-, Fatigue-, Appetite-, or Sweat-Related Issues

Among all included RCTs, only 10 (1.5%) reported sleep-, fatigue-, appetite-, or sweat-related side effects among participants, whereas 674 (98.5%) reported none. The occurrences of side effects reported within these RCTs varied across tertiles of total absolute creatine dose, wherein one (0.4%) of 251 RCTs in the low-dose tertile reported these side effects, compared to none in the moderate-dose tertile and nine of 226 (1.3%) in the high-dose tertile. Chi-square analysis showed a statistically significant association between dose tertile and the occurrence of side effects (χ^2^(2) = 15.007, *p* < 0.001, φc = 0.148). When analyzed by tertiles of total days of supplementation, no sleep-, fatigue-, appetite-, or sweat-related side effects were reported in the low-duration tertile, but four of 199 (2.0%) and six of 215 (2.8%) reported these effects in the moderate- and high-duration tertiles, respectively. Chi-square analysis showed a statistically significant association between duration tertiles and side-effect occurrence (χ^2^(2) = 7.056, *p* = 0.029, φc = 0.102).

#### 3.2.7. Cardiovascular-Related Issues

Among all included RCTs, only six (0.9%) reported cardiovascular-related side effects among participants, whereas 678 (99.1%) reported none. The occurrence of side effects reported within these RCTs varied across tertiles of total absolute creatine dose, with none reported in the low-dose tertile, compared to three (1.4%) of 207 RCTs in the moderate-dose tertile, and three of 2126 (1.3%) in the high-dose tertile. Chi-square analysis showed no association between dose tertile and side-effect occurrence (χ^2^(2) = 3.527, *p* = 0.171, φc = 0.072). When analyzed by tertiles of total days of supplementation, no cardiovascular-related side effects were reported in the low-duration tertile, whereas one of 199 (0.5%) and five of 215 (2.3%) reported these effects in in the moderate- and high-duration tertiles, respectively. Chi-square analysis showed a statistically significant association between duration tertiles and side-effect occurrence (χ^2^(2) = 7.898, *p* = 0.019, φc = 0.107).

#### 3.2.8. Respiratory- or Immune-Related Issues

Among all included RCTs, only one (0.1%) reported respiratory- or immune-related side effects among participants, whereas 683 (99.9%) reported none. These side effects were reported only in the high-dose tertile, with one (0.4%) of the 226 RCTs noting them. Chi-square analysis showed no association between dose tertile and side-effect occurrence (χ^2^(2) = 2.030, *p* = 0.362, φc = 0.054). When analyzed by tertiles of total days of supplementation, no respiratory or immune-related side effects were reported in the low- and moderate-duration tertiles; one of 215 (0.5%) reported these effects in the high-duration tertile. Chi-square analysis showed no significant association between the duration tertile and the occurrence of side effects (χ^2^(2) = 2.185, *p* = 0.335, φc = 0.057).

#### 3.2.9. Psychiatric/Nervous-Related Issues

Among all included RCTs, there were no reported psychiatric or nervous-related side effects among participants in any of the dosing or duration tertiles.

#### 3.2.10. Side Effects Considered as Other

Among all included RCTs, only seven (1.0%) reported respiratory- or immune-related side effects among participants, whereas 677 (99.0%) reported none. The occurrences of side effects reported within these RCTs varied across tertiles of total absolute creatine dose, wherein one (0.4%) of 251 RCTs in the low-dose tertile reported these side effects, compared to two (1.0%) of 207 RCTs in the moderate-dose tertile and four of 226 (1.8%) in the high-dose tertile. Chi-square analysis showed no association between dose tertile and the occurrence of side effects (χ^2^(2) = 2.218, *p* = 0.330, φc = 0.057). When analyzed by tertiles of total days of supplementation, psychiatric or nervous-related side effects were reported in the low (one of 270; 0.5%), moderate (four of 199; 2.0%), and high (two of 215; 0.9%) tertiles. Chi-square analysis showed no significant association between duration tertile and side-effect occurrence (χ^2^(2) = 3.068, *p* = 0.216, φc = 0.067).

#### 3.2.11. Individual Side Effects

Among all included RCTs, when considering the 35 individual side effects noted (i.e., not collapsed to one category as noted above), the presence of the following side effects was noted in RCTs for the dose and duration tertiles, with more reports generally being noted in the higher-dose and longer-duration tertiles: “gastrointestinal distress,” “vertigo,” “headaches,” “dizziness,” “light-headedness,” “nausea,” “diarrhea,” “impaired concentration,” “muscle cramps,” sleep, appetite,” “fatigue,” “sweating,” “edema,” “palpitations,” “thromboembolic events,” “kidney-related,” “liver related,” “respiratory-related,” “pregnancy-related,” and “other.” These results are displayed in Table 3. Although 35 individual side-effect categories were analyzed, not all outcomes were consistently observed across the included RCTs. Some side effects were rarely reported or were limited to certain dose or duration groups, resulting in zero counts in some cases.

### 3.3. Logistic Regression

#### 3.3.1. Covariates-Only Model

In adjusted logistic regression analyses across the RCTs, creatine supplementation was not associated with the odds of reporting side effects (OR = 0.936, 95% CI: 0.670–1.308, *p* = 0.698), dizziness (OR = 1.378, 95% CI: 0.311–6.112, *p* = 0.673), light-headedness (OR = 2.543, 95% CI: 0.155–41.755, *p* = 0.513), diarrhea (OR = 1.306, 95% CI: 0.425–4.009, *p* = 0.641), impaired concentration (OR = 0.462, 95% CI: 0.030–7.086, *p* = 0.579), sleep (OR = 2.311, 95% CI: 0.373–14.328, *p* = 0.368), appetite (OR = 2.036, 95% CI: 0.241–17.210, *p* = 0.514), fatigue (OR = 4.389, 95% CI: 0.462–41.734, *p* = 0.198), edema (OR = 5.949, 95% CI: 0.631–56.074, *p* = 0.119), thromboembolic events (OR = 1.624, 95% CI: 0.138–19.357, *p* = 0.697), kidney-related effects (OR = 1.441, 95% CI: 0.222–9.349, *p* = 0.702), liver-related effects (OR = 0.800, 95% CI: 0.037–17.196, *p* = 0.887), respiratory-related effects (OR = 0.500, 95% CI: 0.019–12.898, *p* = 0.676), and other (OR = 1.208, 95% CI: 0.358–4.077, *p* = 0.761).

Creatine supplementation was associated with the odds of reporting a gastrointestinal issue (OR = 2.583, 95% CI: 1.574–4.238, *p* < 0.001), headache (OR = 3.199, 95% CI: 1.005–10.187, *p* = 0.049), nausea (OR = 2.474, 95% CI: 1.031–5.940, *p* = 0.043), and muscle cramps (OR = 3.309, 95% CI: 1.295–8.457, *p* = 0.012). Odds ratios could not be reliably estimated for the following categories due to sparse data or quasi-complete separation: vertigo, sweating, palpitations, neoplasm-related, nerve/muscle-related, infection-related, renal or urinary-related, psychiatric-related, injury-related, cardiovascular-related, bone-related, metabolic/nutrition-related, skin-related, eye-related, and reproductive-related.

#### 3.3.2. Dose- and Duration-Added Models

In multivariable logistic regression analysis, after adjusting for biological sex, age group, population/fitness level, and health status, the dose tertile showed a trend toward association with the outcome (Wald χ^2^(2) = 5.57, *p* = 0.062). The high-dose tertile was related to greater odds of the outcome compared to the low-dose tertile (OR = 3.32, 95% CI: 1.16–9.48, *p* = 0.025), whereas the moderate-dose tertile was not significantly linked (*p* = 0.345). The tertiles of duration were not associated with the outcome (*p* = 0.867). In the adjusted models, headache, nausea, and muscle cramps were not associated with dose tertile, duration tertile, or other characteristics (all *p* > 0.10).

### 3.4. GLM Analysis

**Figure 2:**
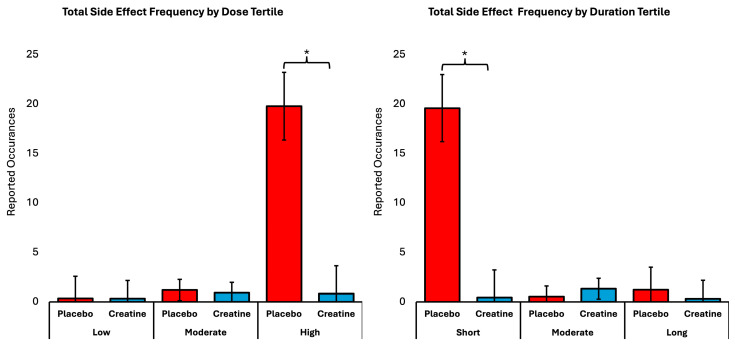
Total side effect frequency in dose and duration tertiles. Total number of reported side effects per randomized controlled trial (mean ± SD), stratified by creatine supplementation group (creatine vs. placebo) across tertiles of total dose (**left panel**) and supplementation duration (**right panel**). Higher-dose and longer-duration tertiles were associated with greater overall side effect reporting at the study level; however, placebo groups frequently reported side effect frequencies comparable to or higher than those in the highest exposure tertiles. Asterisks indicate significant between-group differences within tertiles (*p* < 0.05).

In addition to the binary study-level analyses, we performed generalized linear modeling based on the total count of reported adverse events in each study arm. Although this method offers more detail than binary data, the information is still aggregated at the study-arm level and does not reflect individual participant risk modeling. The overall GLM multivariate analysis revealed statistically significant Wilks’ Lambdas for the dose (*p* = 0.029, η^2^ = 0.028), duration (*p* = 0.021, η^2^ = 0.028), supplement × dose (*p* = 0.048, η^2^ = 0.027), and supplement × duration (*p* = 0.009, η^2^ = 0.030). Univariate analysis revealed that the creatine and placebo study arms of the RCTs differed in the total frequency of side effects reported across the tertile doses (*p* < 0.001, η^2^ = 0.046) and durations (*p* < 0.001, η^2^ = 0.044), as well as by supplement × dose (*p* < 0.001, η^2^ = 0.046) and by supplement × duration (*p* < 0.001, η^2^ = 0.051). Pairwise comparisons found that participants in the high-dose tertile reported a higher total frequency of side effects than those in the low (9.965 counts, [7.308, 12.621], *p* < 0.001) and moderate (9.240 counts, [6.896, 11.584], *p* < 0.001) tertiles. Regarding study duration, low-tertile RCT participants reported more side effects than the moderate (9.076 counts, [6.749, 11.403], *p* < 0.001) and high (9.241 counts, [6.592, 11.890], *p* < 0.001) tertiles. In terms of supplement × dose, those consuming the placebo reported a higher total frequency of side effects across RCTs than those taking creatine (18.959 counts, [14,525, 23.393], *p* < 0.001) in the high-dose tertile. Lastly, with respect to study duration, participants receiving a placebo reported a higher total frequency of side effects across RCTs than those taking creatine (19.140 counts, [14.739, 23.540], *p* < 0.001) in the low-duration tertile. Figure 2 displays the total side effect frequency in the dose and duration tertiles.

## 4. Discussion

The primary purpose of this analysis was to systematically examine the prevalence, distribution, and determinants of side effects associated with CrM and to assess whether the total CrM dose or the duration of exposure predicts side effects, which were classified by physiological system. The goal was to provide a more detailed, clinically meaningful evaluation of CrM safety across different dosing regimens and supplementation periods. This comprehensive analysis of 684 RCTs involving 1337 participants found that: (1) the overall reporting rate for side effects in creatine studies was low; (2) side effects are more likely to be reported in trials with higher doses and/or longer durations; and (3) after accounting for key study variables (e.g., biological sex and age), CrM did not statistically significantly increase the risk of side effects in most physiological systems. Although higher exposure tertiles were associated with a greater proportion of studies reporting at least one adverse event, the effect sizes were small, and the absolute frequency of reported events remained low. It is important to note that side-effect reporting across multiple outcome categories was limited to only a few trials, resulting in highly imbalanced data dominated by studies with zero events. These patterns of sparse events naturally constrained the size of the effect estimates and should be considered when interpreting the statistically significant results. Crucially, conclusions about creatine safety were based on overall reporting trends, effect size, and the absence of consistent exposure–response relationships across different models, rather than on isolated odds ratios from infrequent outcomes. Additionally, no clear monotonic exposure–response gradient was observed across tertiles. Therefore, the primary safety conclusions relied on overall reporting patterns and effect magnitudes rather than on isolated, statistically significant findings from sparse categories. Nevertheless, these findings corroborate those of Kreider and colleagues [5] and Longobardi and colleagues [8], further supporting the favorable safety profile of creatine, particularly CrM. It is important to note that we did not include the RCT by Kieburtz et al. [7], which evaluated the effects of 10 g/day of either CrM or placebo on 1741 men and women with early Parkinson’s disease for up to 8 years. This study was not included in the current analysis because the side effects occurred in both the creatine and placebo groups and were not always directly attributable to CrM. Including this trial might have distorted the overall side effect rates due to its large sample size, extended duration, and the specific clinical context of the disease, which are notably different from the included studies. In the Kieburtz et al. [7] study, the prevalence of side effects was generally low across key categories, such as overall side effects (~4–5%) and nervous/nerve and muscle complaints (~3–4%). Differences between the creatine and placebo groups were minimal, typically less than 1% point and not statistically significant (Reference Table 3 of Kreider et al. [5]). Although the larger sample size and longer duration of this study resulted in more reported events, the associated odds ratios and effect sizes did not indicate a meaningful increase in creatine-related side effects.

Although early research indicated no side effects from CrM supplementation at doses of up to 25 g/day for 84 days in American football players during intense training [9], subsequent media reports suggested side effects, including cramping, dehydration, musculoskeletal injuries, gastrointestinal problems, and kidney concerns. However, further studies showed no increased rates of musculoskeletal injuries [10], dehydration [11,12,13,14,15,16], muscle cramps [12,17,18], gastrointestinal symptoms [5], renal problems [19,20,21,22,23,24,25,26,27,28,29], or long-term side effects [28,30,31]. Supporting these findings, Kreider [32] noted that no controlled studies have confirmed the side effects reported in the media. Consequently, the International Society of Sport Nutrition’s 2007 [33] and 2017 [1] position stands affirm that CrM is safe and effective for all age groups, although they caution that restrictions on creatine use may unintentionally increase athletes’ risk. Building on this literature, the current dose- and duration-based tertile analyses show that CrM does not exhibit a dose- or time-dependent increase in side effects involving most physiological systems, reaffirming its safety across various supplementation paradigms.

### 4.1. Overall Safety Profile of Creatine Supplementation

Across all included RCTs, less than 14% reported any side effects in the creatine study arm, with most trials reporting none. These results support the long-held view that creatine supplementation, when used in controlled clinical settings, is generally well-tolerated across diverse populations, dosages, and durations [1,5,32,33]. Previous work has demonstrated that reported side effects in RCTs were similar between the placebo (13.2%) and creatine (13.7%) study arms, with no significant difference between the two (*p* = 0.776) [5]. Notably, the current findings build on previous safety summaries by incorporating analyses of dose and duration across a diverse set of studies. The very low incidence of reported side effects across these trials is noteworthy, particularly given the inclusion of clinical populations, older adults, and long-term interventions. This suggests that concerns about widespread or serious side effects of creatine may be overstated given the overall volume of human clinical data. Despite this evidence, myths and misconceptions are likely to persist across various platforms (e.g., social media), and it is paramount that the scientific community widely disseminates these findings to address these concerns [32].

### 4.2. Dose and Duration Effects on Side Effect Reporting

Although the overall incidence of reported side effects was low, studies with higher doses and longer supplementation periods were more likely to report at least one side effect compared to those with lower doses and shorter durations. These links were modest (Cramér’s φ ≈ 0.14–0.16) and consistent across different physiological systems, indicating a study-level dose–exposure relationship rather than dose-dependent toxicity. Notably, most randomized controlled trials in the highest exposure tertiles reported no side effects, suggesting that greater cumulative exposure does not necessarily lead to frequent or severe harm. The linear pattern observed across dose categories supports a graded exposure–response relationship, likely due to increased reporting opportunities rather than a threshold effect. Overall, these results indicate that creatine monohydrate was not associated with a consistent or clinically meaningful increase in side-effect reporting compared to placebo at the study level, even with long-term use.

### 4.3. Physiological Classification of Side Effects

Gastrointestinal issues were the most frequently reported side effect, followed by musculoskeletal-, neurological/vestibular-, and sleep- or fatigue-related concerns, consistent with prior work on creatine safety [1,5,32,33]. Most notably, gastrointestinal distress is a commonly reported side effect, and those supplementing with creatine may need to divide the dose into smaller boluses to alleviate it [34]; however, it is worth noting that this side effect was not persistent [35]. Even within these side effect categories, incidence rates were generally low, typically below 10% in RCTs, further supporting the overall safety profile of creatine [5]. Side effects affecting the kidneys, liver, heart, lungs, and mental health were very rare, with many categories appearing in fewer than 1% of studies. The few reports of renal and hepatic side effects across hundreds of RCTs are especially noteworthy given the common misconception that creatine causes kidney or liver problems. In this review, only three trials reported renal or urinary side effects, all in the highest-dose group, and no clear association with duration was observed. These findings support existing mechanistic and clinical evidence indicating that creatine does not impair kidney function in healthy individuals or patients when used at recommended doses [5,8,36,37].

### 4.4. Logistic Regression Findings and Interpretation

Adjusted logistic regression analyses offer additional context for these prevalence results. After adjusting for biological sex, age group, fitness level, and health status, creatine supplementation generally did not increase the likelihood of reporting side effects across most physiological systems. Notably, in covariate-only models, creatine was associated with higher odds of gastrointestinal symptoms, headaches, nausea, and muscle cramps. However, these findings should be viewed cautiously for several reasons. First, the models reflect study-level reporting rather than individual participant incidence. An increased odds ratio indicates at least one report in a trial, not necessarily a higher individual risk. Second, many side effects were rare, leading to wide confidence intervals and unstable estimates. Third, when dose and duration tertiles were included, some associations weakened or disappeared, implying that exposure level and study design influence side effect reporting. Models including dose and duration showed a trend toward higher odds in the high-dose group, but no consistent link was observed with duration tertiles. This suggests that overall exposure may affect reporting, whereas duration alone does not independently predict side effects when dose is considered.

### 4.5. Frequency-Based Analyses and Complementary Evidence

Frequency-based GLM analyses serve as a valuable complement to safety interpretation by providing an alternative perspective. While prevalence analyses indicate whether any side effect was reported, frequency analyses count the total number of reported side effects per study. These models revealed that higher-dose and longer-duration groups reported more side effects overall; however, placebo groups often reported as many or more side effects than the creatine groups, particularly in high-dose and long-duration groups, further corroborating the findings of Kreider et al. [5]. This pattern suggests that many reported side effects may reflect nonspecific symptoms or background reporting rather than clear supplement-attributable events. Nevertheless, it is important to consider other possible explanations. Variations in how intensively adverse events are monitored, differences in study population characteristics, the length of follow-up periods, and the criteria for reporting events across trials can all affect the reported event rates in both the creatine and placebo groups. Consequently, higher event frequencies in placebo groups should not be taken as conclusive proof of nocebo effects; instead, they highlight the complex methodological differences inherent in combined RCT analyses. The consistent pattern seen across various dose and duration groups further undermines the idea that creatine causes side effects.

### 4.6. Limitations and Strengths

Several methodological points warrant discussion. First, selecting a study-level analytic framework was deliberate to enable consistent comparisons across trials. While this approach does not estimate individual risk levels, it effectively identifies reporting trends and safety signals in the literature. Although we performed additional GLM analyses using the total frequency of reported adverse events within each RCT arm, the data were still aggregated at the trial level. As a result, the findings indicate reporting patterns across studies and should not be taken as direct estimates of individual participant risk. Additionally, each RCT arm was given equal weight in the analysis regardless of sample size, which could affect accuracy when comparing heterogeneous trials. Differences in adverse event definitions and reporting procedures across studies may also lead to reporting bias. Lastly, modeling rare events at the study level may reduce stability in some tertile comparisons. Therefore, the conclusions should be viewed as safety trends at the study level rather than definitive individual exposure–response relationships.

Each RCT arm contributed equally to the analyses regardless of sample size. This approach maintains methodological consistency and prevents larger trials from having disproportionate influence, but it does not weight estimates by participant exposure volume. Consequently, comparisons between studies with significantly different enrollment sizes might be less precise. Future studies using inverse-variance weighting or participant-level meta-analytic models could further refine the results. Variability in adverse event reporting across trials is another key factor. Reporting depended on the methodological rigor, monitoring procedures, definitions, and thresholds used in individual RCTs. These differences can affect the observed frequencies and introduce heterogeneity in reporting across studies. Additionally, modeling rare events at the study level may cause statistical instability, especially within tertile stratifications where event counts are low. Therefore, null results in low-frequency categories should not be seen as conclusive evidence of no risk but rather as due to limited event data.

An additional methodological point concerns how exposure was estimated. When participant body mass data was unavailable, estimated values were used to approximate the relative dose exposure. This method allowed for the inclusion of all RCT evidence and helped standardize the dose calculations across trials, but it might cause some misclassification of exposure. Such misclassification is likely nondifferential, which tends to bias results toward the null; however, precise individual dose normalization cannot be assured. Additionally, dose and duration variables were divided into tertiles to improve clarity and minimize the impact of outliers. While this enhances understanding and consistency across diverse studies, it also reduces the amount of detail and may weaken statistical power compared to treating exposure as a continuous variable. As a result, subtle dose–response relationships, if any, might not be fully identified through tertile categorization. Then, limited data and near-complete separation hampered the interpretation of certain rare side effects, underscoring their infrequency in creatine studies. Notable strengths of this analysis include the comprehensive inclusion of trials; systematic categorization of side effects; combined assessment of prevalence, odds, and frequency; and conservative interpretation of findings. Accordingly, conclusions should be interpreted within the constraints of an ecological study-level analysis and should not be construed as definitive estimates of individual risk.

## 5. Conclusions

These findings suggest that creatine supplementation is safe across a range of doses, durations, and populations according to human trials. While higher total doses and longer supplementation periods are associated with more side effects at the study level, the overall incidence remains low, with most effects being mild and nonspecific. Furthermore, placebo groups often report similar or even higher rates of side effects. These results reinforce the consensus on creatine’s safety and add nuance by considering exposure levels and study design factors. Future research should incorporate standardized side effect reporting and individual-level meta-analyses. Based on the current evidence, creatine is one of the most well-studied and well-tolerated dietary supplements. No consistent or clinically meaningful dose-dependent increases in side-effect reporting were observed across models; even at higher doses and prolonged durations, reporting remained low and largely comparable to placebo at the study level. This analysis affirms previous findings on the overall safety of creatine supplementation and suggests that high-dose or longer-duration supplementation is well-tolerated by both clinical and athletic users. Notably, the doses examined exceed the typical 3–5 g/day used by athletes, further affirming the safety of higher intake levels. Future research should focus on conducting participant-level meta-analyses, developing standardized frameworks for adverse event reporting, and designing prospective trials that are specifically powered to assess safety outcomes.

## Figures and Tables

**Table 1:** Studies with participants reporting side effects.

		**Low**	**Moderate**	**High**	**Total**	**Statistic**	
**Dose**	**Count**	22	24	47	93	***p*-Value**	<0.001
	**% within Tertile**	8.8%	11.6%	20.8%	13.6%	**χ^2^**	15.667
	**% of Total**	3.2%	3.5%	6.9%	13.6%	**φc**	0.151
		**Short**	**Moderate**	**Long**			
**Duration**	**Count**	19	31	43	93	***p*-Value**	<0.001
	**% within Tertile**	7.0%	15.6%	20.0%	13.6%	**χ^2^**	18.058
	**% of Total**	2.8%	4.5%	6.3%	13.6%	**φc**	0.162

Values represent the number of randomized controlled trials (RCTs) in which at least one adverse event was reported within the creatine supplementation arm, stratified by tertiles of total creatine dose (top panel) and supplementation duration (bottom panel). Percentages are presented as the proportion of studies within each tertile and as a percentage of the total number of included RCTs (*n* = 684). Associations between tertiles and adverse event reporting were evaluated using Pearson’s chi-square (χ^2^) tests. Effect sizes are reported as Cramér’s V (φc), with values < 0.10 considered weak, 0.10–0.30 considered moderate, and >0.30 considered strong. Abbreviations: χ^2^, chi-square statistic; φc, Cramér’s phi coefficient. *p* < 0.05 indicates statistical significance.

**Table 2:** Study-level prevalence of reported side effects by physiological classification across creatine dose and duration tertiles.

		Dose						Duration					
		Low	Moderate	High	Total	Statistic		Short	Moderate	Long	Total	Statistic	
**Gastrointestinal Issues**	Count	14	17	37	68	***p*-Value**	<0.001	14	22	32	68	***p*-Value**	0.002
	% within Tertile	5.6%	8.2%	16.4%	9.9%	**χ^2^**	16.467	5.2%	11.1%	14.9%	9.9%	**χ^2^**	12.963
	% of Total	2.0%	2.5%	5.4%	9.9%	**φc**	0.155	2.0%	3.2%	4.7%	9.9%	**φc**	0.138
**Renal- or Urinary-Related Issues**	Count	0	0	3	3	***p*-Value**	0.047	0	0	3	3	***p*-Value**	0.037
	% within Tertile	0.0%	0.0%	1.3%	0.4%	**χ^2^**	6.106	0.0%	0.0%	1.4%	0.4%	**χ^2^**	6.573
	% of Total	0.0%	0.0%	0.4%	0.4%	**φc**	0.094	0.0%	0.0%	0.4%	0.4%	**φc**	0.098
**Liver-, Metabolic-, or Nutrition-Related Issues**	Count	0	0	1	1	***p*-Value**	0.362	0	0	1	1	***p*-Value**	0.335
	% within Tertile	0.0%	0.0%	0.4%	0.1%	**χ^2^**	2.03	0.0%	0.0%	0.5%	0.1%	**χ^2^**	2.185
	% of Total	0.0%	0.0%	0.1%	0.1%	**φc**	0.054	0.0%	0.0%	0.1%	0.1%	**φc**	0.057
**Musculoskeletal Issues**	Count	1	5	14	20	***p*-Value**	0.001	0	8	12	20	***p*-Value**	0.001
	% within Tertile	0.4%	2.4%	6.2%	2.9%	**χ^2^**	14.346	0.0%	4.0%	5.6%	2.9%	**χ^2^**	14.324
	% of Total	0.1%	0.7%	2.0%	2.9%	**φc**	0.145	0.0%	1.2%	1.8%	2.9%	**φc**	0.145
**Neurological- or Vestibular-Related Issues**	Count	3	3	11	17	***p*-Value**	0.019	1	7	9	17	***p*-Value**	0.015
	% within Tertile	1.2%	1.4%	4.9%	2.5%	**χ^2^**	7.931	0.4%	3.5%	4.2%	2.5%	**χ^2^**	8.424
	% of Total	0.4%	0.4%	1.6%	2.5%	**φc**	0.108	0.1%	1.0%	1.3%	2.5%	**φc**	0.111
**Sleep-, Fatigue-, Appetite-, or Sweat-Related Issues**	Count	1	0	9	10	***p*-Value**	0.001	0	4	6	10	***p*-Value**	0.029
	% within Tertile	0.4%	0.0%	4.0%	1.5%	**χ^2^**	15.007	0.0%	2.0%	2.8%	1.5%	**χ^2^**	7.056
	% of Total	0.1%	0.0%	1.3%	1.5%	**φc**	0.148	0.0%	0.6%	0.9%	1.5%	**φc**	0.102
**Cardiovascular-Related Issues**	Count	0	3	3	6	***p*-Value**	0.171	0	1	5	6	***p*-Value**	0.019
	% within Tertile	0.0%	1.4%	1.3%	0.9%	**χ^2^**	3.527	0.0%	0.5%	2.3%	0.9%	**χ^2^**	7.898
	% of Total	0.0%	0.4%	0.4%	0.9%	**φc**	0.072	0.0%	0.1%	0.7%	0.9%	**φc**	0.107
**Respiratory- or Immune-Related Issues**	Count	0	0	1	1	***p*-Value**	0.362	0	0	1	1	***p*-Value**	0.335
	% within Tertile	0.0%	0.0%	0.4%	0.1%	**χ^2^**	2.03	0.0%	0.0%	0.5%	0.1%	**χ^2^**	2.185
	% of Total	0.0%	0.0%	0.1%	0.1%	**φc**	0.054	0.0%	0.0%	0.1%	0.1%	**φc**	0.057
**Psychiatric/Nervous-Related Issues**	Count	0	0	0	0	***p*-Value**	-	0	0	0	0	***p*-Value**	-
	% within Tertile	0.0%	0.0%	0.0%	0.0%	**χ^2^**	-	0.0%	0.0%	0.0%	0.0%	**χ^2^**	-
	% of Total	0.0%	0.0%	0.0%	0.0%	**φc**	-	0.0%	0.0%	0.0%	0.0%	**φc**	-
**Side Effects Considered as Other**	Count	1	2	4	7	***p*-Value**	0.330	1	4	2	7	***p*-Value**	0.216
	% within Tertile	0.4%	1.0%	1.8%	1.0%	**χ^2^**	2.218	0.4%	2.0%	0.9%	1.0%	**χ^2^**	3.068
	% of Total	0.1%	0.3%	0.6%	1.0%	**φc**	0.057	0.1%	0.6%	0.3%	1.0%	**φc**	0.067

Values represent the number of randomized controlled trials (RCTs) in which at least one participant in the creatine supplementation arm reported an adverse event within each physiological category, stratified by tertiles of total creatine dose and supplementation duration. Percentages are shown as the proportion of studies within each tertile (% within tertile) and as a proportion of all included RCTs (% of total; *n* = 684). Associations between dose or duration tertiles and adverse event reporting were assessed using Pearson’s chi-square (χ^2^) tests. Effect sizes are reported as Cramér’s phi (φc), with larger values indicating stronger study-level associations. Blank or dashed entries indicate no reported events within that category. Abbreviations: χ^2^, chi-square statistic; φc, Cramér’s phi coefficient.

**Table 3:** Prevalence of reported side effects by physiological classification across creatine dose and duration tertiles in individual categories.

	Dose							Duration						
	Low	Moderate	High	Total	*p*-Value	χ^2^	φc	Short	Moderate	Long	Total	*p*-Value	χ^2^	φc
Gastrointestinal/Abdominal	12	17	33	62	0.001	14.177	0.144	13	20	29	62	0.004	11.255	0.128
Vertigo	0	1	2	3	0.342	2.146	0.056	0	0	3	3	0.037	6.573	0.098
Headache	3	2	8	13	0.086	4.896	0.085	1	7	5	13	0.041	6.390	0.097
Dizziness	1	1	3	5	0.435	1.666	0.049	0	2	3	5	0.174	3.502	0.072
Light-Headed	1	0	1	2	0.645	0.878	0.036	0	1	1	2	0.519	1.313	0.044
Nausea	2	4	12	18	0.007	10.019	0.121	1	7	10	18	0.009	9.420	0.117
Diarrhea	0	1	7	8	0.004	11.080	0.127	0	0	8	8	0.000	17.658	0.161
Impaired Concentration	0	0	1	1	0.362	2.030	0.054	0	0	1	1	0.335	2.185	0.057
Muscle Cramping/Pain	1	5	14	20	0.001	14.346	0.145	0	8	12	20	0.001	14.324	0.145
Sleep Disturbances †	1	0	3	4	0.173	3.512	0.072	0	2	2	4	0.268	2.634	0.062
Poor Appetite †	0	0	3	3	0.047	6.106	0.094	0	0	3	3	0.037	6.573	0.098
Fatigue	0	0	4	4	0.017	8.154	0.109	0	2	2	4	0.268	2.634	0.062
Excessive Sweating	0	0	2	2	0.131	4.065	0.077	0	0	2	2	0.112	4.376	0.080
Edema	0	3	2	5	0.183	3.394	0.070	0	1	4	5	0.052	5.911	0.093
Palpitations	0	0	1	1	0.362	2.030	0.054	0	0	1	1	0.335	2.185	0.057
Thromboembolic Events	0	0	2	2	0.131	4.065	0.077	0	0	2	2	0.112	4.376	0.080
Kidney-Related	0	0	3	3	0.047	6.106	0.094	0	0	3	3	0.037	6.573	0.098
Liver-Related	0	0	1	1	0.362	2.030	0.054	0	0	1	1	0.335	2.185	0.057
Respiratory †	0	0	1	1	0.362	2.030	0.054	0	0	1	1	0.335	2.185	0.057
Pregnancy	0	1	0	1	0.315	2.308	0.058	0	0	1	1	0.335	2.185	0.057
Other	1	1	4	6	0.212	3.103	0.067	1	4	1	6	0.125	4.155	0.078

This table summarizes the number of randomized controlled trials (RCTs) reporting each individual adverse event (yes/no) across creatine dose tertiles (low, moderate, and high) and supplementation duration tertiles (short, moderate, and long). For each adverse event category, chi-square (χ^2^) tests were used to evaluate associations between tertile classification and adverse event occurrence, with corresponding *p*-values and φc (Cramér’s V) reported as indices of effect size. Overall, adverse event reporting was low across all categories; however, select gastrointestinal and musculoskeletal events were more frequently reported in higher-dose and longer-duration tertiles. † Indicates side effects reported exclusively in a single long-term clinical trial of 1741 patients with Parkinson’s disease consuming 10 g·day^−1^ of placebo or creatine monohydrate for up to 8 years [7].

## Data Availability

No new data were created or analyzed in this study. Data sharing is not applicable to this article.

## Abbreviations

The following abbreviations are used in this manuscript:

φc	Cramér’s Effect Size
SEs	Side effects
CrM	Creatine monohydrate
CIs	Confidence interval
GLMs	Generalized linear models
GRAS	Generally Recognized as Safe
OR	Odds ratio
η^2^	Partial eta squared
RCT	Randomized controlled trial
U.S.	United States of America
